# Characterizing individual variability in mussel (*Mytilus galloprovincialis*) growth and testing its physiological drivers using Functional Data Analysis

**DOI:** 10.1371/journal.pone.0205981

**Published:** 2018-10-18

**Authors:** Isabel Fuentes-Santos, Uxío Labarta, María José Fernández-Reiriz

**Affiliations:** Consejo Superior de Investigaciones Científicas (CSIC), Instituto de Investigaciones Marinas (IIM), C/Eduardo Cabello 6, Vigo, Spain; University of Waikato, NEW ZEALAND

## Abstract

Determining the magnitude and causes of intrinsic variability is a main issue in the analysis of bivalve growth. Inter-individual variability in bivalve growth has been attributed to differences in the physiological performance. This hypothesis has been commonly tested comparing the physiological rates of fast and slow growers after size differentiation has occurred. This experimental design may detect a link between growth and physiological performance, but we cannot interpret the posterior physiological performance as a driver for the prior growth variability. Considering these limitations, this work introduces a new methodological framework for the analysis of bivalve growth variability. We have conducted sequential measurements of size and physiological performance (feeding, digestion and metabolic rates) in even-sized mussels growing under homogeneous environmental conditions. This experimental design allows us to distinguish between changes over time within individuals, i.e. growth and trends in the physiological rates, from differences between individuals with respect to a baseline level. In addition, Functional Data Analysis provides powerful tools to summarize all the information obtained in the exhaustive sampling scheme and to test whether differences in the physiological performance enhance growth dispersion. Our results report an increasing dispersion in both size and physiological performance over time. Although mussels grew during the experiment, it is difficult to detect any increasing or decreasing temporal pattern in their feeding, digestion and metabolic rates due to the large inter-individual variability. Comparison between the growth and physiological patterns of mussels with final size above (fast growers) and below (slow growers) the median found that fast growers had larger feeding and digestion rates and lower metabolic expenditures during the experimental culture than mussels with slow growth, which agrees with the hypothesis of a physiological basis for bivalve growth variability.

## 1 Introduction

Bivalve growth is subjected to great variability due to multiple intrinsic (i.e. genetic), environmental (temperature, food), and social (competition with conspecifics) factors. Understanding the nature and contribution of these factors to growth would provide insight into their population dynamic [1] and consequently, helps in the development of aquaculture management and sustainability plans, which have been increasingly demanded in areas that support large aquaculture production, such as the Galcian Rías (NW Spain) [2].

The effect of environmental conditions on bivalve growth has been extensively studied [3,4]. Several studies have addressed the effects of inter and intra-specific competition on mussel growth and survival [5–9]. Intrinsic variability, i.e. heterogeneous growth of even-aged individuals under the same environmental conditions, has received less attention, although overlooking this variability may result in incorrect predictions of population dynamics [1,10].

Bayne (1999) [11] pointed out that the endogenous variability of bivalve growth can be explained through the physiological components integrated in the energy budged. Following this proposal, recent works have analyzed the physiological performance of individuals with fast and slow growth in even-aged populations of oysters, *Crassostrea virginica* [12] and *Crassostrea gigas*, [13], clams *Ruditapes philippinarum* [14–16], and mussels *Mytilus galloprovincialis* [17–19]. In agreement with the hypothesis proposed by [11], these works found a link between individual differences in growth and the rates of food acquisition, absorption and metabolic costs.

The studies outlined above followed a cross-sectional design, which measures the response of different groups of individuals at a single time point, to test the physiological basis of growth hetereogenity. They compare the physiological performance of even-aged bivalves that reached small and large sizes after a growing period under common environmental conditions. This experimental design has two important limitations. Although their aim is to determine the causes of individual growth variability, they compare the performance of two groups in a single cohort based on a single measure of each individual, while repeated measures of the same individual are required for a proper characterization of individual growth. Some studies have pointed out this issue and proposed mixed effects models as a tool for including individual variability in the estimation of growth at the population level [1,10,20]. On the other hand, most of these works measure the physiological performance after the size differentiation has occurred and compare feeding (clearance rate), digestion (absorption efficiency) and metabolic (respiration and excretion) rates between growth groups [14–16,18,19]. As the physiological experiment are conducted when fast growers are larger than slow growers, these studies test whether feeding and metabolic rates depend on size, thus any observed difference between groups is a consequence not a driver of the prior growth variability. To test which physiological components of the energy budget enhance growth variability, these factors should be measured during growth

This work addresses individual growth variability from a different methodological perspective in order to avoid the shortcuts outlined above. For this purpose, we have used a new experimental design, as well as new statistical techniques. We have monitored the individual growth and physiological performance of even-aged and even-sized mussels in the laboratory. This experimental design shall allow us to measure growth variability and to link physiological and growth performance. The recently developed Functional Data Analysis [21,22], where each data is a curve recording the growth (in terms of shell length and live weight) and physiological components of the energy budget of mussels during the experiment, where applied to characterize individual growth variability, and find a link between physiological and growth performance.

## 2 Methods

### 2.1 Ethics statement

No specific permissions were required for the sampling of mussel *Mytilus galloprovincialis*. The biological samples used in this work (mussels) belonged to a private enterprise that cultivates mussels in Galicia, NW Spain: Promotora Industrial Sadense (PROINSA Mussel Farm). We confirm that the field study did not involve endangered or protected species and was not performed in a national park or other protected area.

### 2.2 Experimental design

Juvenile mussels (*Mytilus galloprovincialis*), from the same spawning event in the spring of 2016, were gathered the 4^th^ November 2016 from artificial collector ropes suspended on a commercial mussel raft (subtidal environment) in the Ría de Ares-Betanzos (NW Spain). 160 even-sized individuals with shell length L = 20mm and mean total fresh weight TFW = 0.66g (sd = 0.089) were moved to the laboratory and placed in individual labeled recipients inside 4 flow-through plastic aquaria (44.5x40x14.5 cm, 19 l seawater volume), each aquarium contained 40 mussels. We have 3 experimental aquaria while mussels in the fourth tank were used to replace those sacrificed in each sampling to maintain the population of the experimental aquaria constant. We selected 48 mussels, 16 per experimental aquarium, to monitor their growth and physiological performance through monthly samplings during the experimental culture that lasted five months, up to the 25^th^ April 2017, none of the 48 mussels died during the experimental culture. In parallel, a sample of 16 mussels with the same size distribution as those monitored was sacrificed every month to determine their morphometric characteristics, dry weight and gill area. We focus on analyzing the data obtained through individual monitoring, and shall not go deeper on the additional information provided by the parallel sampling.

### 2.3 Diet characteristics

Mussels were fed with a mixture of R. lens and freeze-dried sediment (proportion 70:30 w:w) supplied in a continuous flow to each aquarium by a peristaltic pump (ISMATEC MPC Process) to maintain a concentration of 10,000 cells/ml. The diet was maintained in an aerated tank of 60 l to generate a homogeneous mixture and prevent sedimentation. R. lens maximizes the feeding, digestion and the assimilatory balance of nutrients and energy in mussel M. galloprovincialis compared to other classical monoalgal diets [23]. Sediment from a mussel raft area in the Ria de Vigo was washed, frozen, lyophilized and filtered through a 50 μm mesh. Sediment (91% inorganic content) was incorporated to the diet as an inorganic tracer is required during the measurements of the absorption efficiency [24].

### 2.4 Sequential samplings

The growth and physiological performance of each one of the 48 mussels were measured every month from 14/11/2016 to 25/04/2017. Shell length was recorded up to the nearest 0.1 mm with a caliper, and total fresh weight was measured up to the nearest mg with a Sartorius micro M3P analytical balance.

Triplicate samples of the diet were taken in each physiological experiment to determine its particulate characteristics following the same protocol as [18]. Samples were filtered onto Whatman GF/C fiberglass filters that had been previously washed, ashed and weighed. Filters were rinsed with a solution of ammonium formate 0.5 M to remove sea salt. Filtered samples were oven-dried at 100°C until constant weight to determine the total particulate matter (TPM, mg/l), and then ashed at 450°C in a muffle furnace to determine the particulate inorganic matter (PIM, mg/l), particulate organic matter (POM, mg/l) was calculated as the difference between TPM and PIM. The particulate organic matter (POM) ranked between 0.673 and 0.734 mg/l with constant values for each tank and experiment, and the organic content of the diet (POM/TPM) ranked from 0.72 to 0.75.

The clearance rate (CR, l/h) was estimated by the flow-through procedure described in [25]. Each mussel was placed in an experimental chamber and its clearance rate determined as the reduction in suspended particle concentration between water inflow and outflow of the chamber, measured in duplicate with an electronic Multisizer Coulter II counter with a 100-μm orifice. The organic ingestion rate (OIR, mg/h) was obtained as the product of CR by POM. The absorption efficiency (AE) was estimated from the organic fraction of the ingested food and mussels`feces, following the method of [24]. The absorption rate (AR, mg/h) was calculated as the product of AE by OIR.

Respiration rates (VO_2_, mlO_2_/h) were estimated measuring the O_2_ decline in individual respirometers filled with filtered seawater (1 μm). O_2_ concentration was monitored with YSI58 oxygen meters connected to YSI5730 probe while it was above the 30% of the initial concentration. O_2_ decline was estimated as the difference between the initial and final concentration. Ammonia excretion rates (VNH_4_-N, μgNH_4_-N/h) were estimated after incubating mussels in individual experimental chambers filled with filtered seawater (0.2 μm). NH_4_-N concentration in the water was measured through the phenol-hypochlorite method [26]. Ammonia excretion rates were determined as the difference in ammonia concentration between the experimental and two empty control chambers.

The Scope for Growth (SFG, J/h) is defined as the fraction of the absorbed energy available for somatic and gametogenic growth once metabolic requirements were met [27]. The SFG was computed through the balance equation SFG = AR—M [28,29], i.e. as the difference between the assimilated energy (AR) and the metabolic costs (M), comprising both respiration and excretion rates.

### 2.5 Statistical analysis

Kernel density estimation with plug-in bandwidth [30,31] was used to fit the size distribution of mussels in terms of shell length (L, mm) and total fresh weight (TFW, g) across samplings. One-factor ANOVA by ranks followed by post-hoc Tukey-HSD tests were applied to test whether the size and physiological rates of mussels varied across samplings. A nonparametric distance-based test for homogeneity of dispersions [32] followed by post-hoc Tukey-HSD tests was applied to test whether the dispersion of the size distributions and physiological rates increased during the culture period.

The growth and physiological performance of mussels over the experimental culture were characterized through the recently developed techniques for the analysis of functional data. In Functional Data Analysis (FDA) [21] we assume that a vector, *Y* = (*y*_1_,…,*y*_*n*_) = (*Y*(*t*_1_),…,*Y*(*t*_*n*_)), represents a set of discrete observations of a continuous function, *Y*(*t*). This method replaces the sampled functions (discrete observations) by functional representations (curves). In our case, each curve represents the growth, feeding and metabolic performance of a mussel during the experimental culture. FDA allows working with irregular sampling intervals and missing values.

Functional representations of the growth and performance of mussels were obtained by non-parametric smoothing. We assume that the functional data *Y*(*t*) is observed through a model, Y(*t*_*i*_) = *X*(*t*_*i*_) + *e*(*t*_*i*_), where the residuals *e*(*t*) are independent of the model term, *X*(*t*). The nonparametric estimate of *X*(*t*) is obtained as follows

$$X(t)=\frac{\sum_{j=1}^{n}y_{j}K_{h}(t-t_{j})}{\sum_{j=1}^{n}K_{h}(t-t_{j})}$$

where *K*_*h*_(*t*) = *h*^−1^*K*(*t*/*h*) is the Gaussian kernel with bandwidth parameter, *h*, estimated by generalized cross validation [21]. Mussels with extreme growth patterns were identified through a functional outliers detection algorithm based on the depth of each curve with respect to the 10% trimmed mode [33], depth measures provide a center-outward ordering of the set of curves indicating how close to the center of the functional distribution is each curve. One-factor functional ANOVA [34] was applied to test whether slow and fast growing individuals had different physiological and metabolic patterns.

Data analysis was performed with the statistical software R.3.4.1 [35]. We used the *ks* package [36] for kernel density estimation, and the *fda*.*usc* package [37] for functional data analysis.

## 3 Results

### 3.1 Individual growth and performance

**Fig 1:**
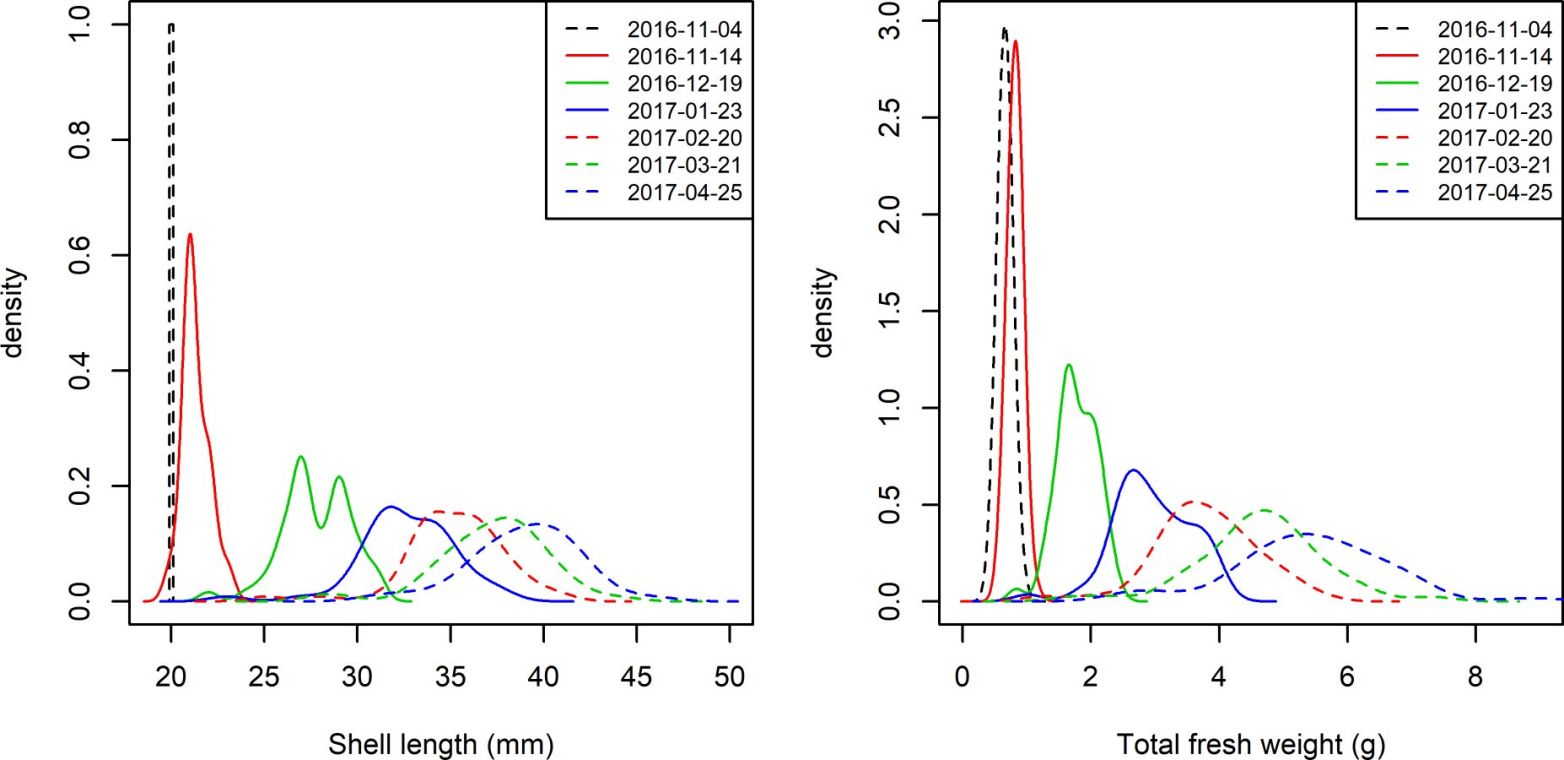
Distribution of shell length (left) and total fresh weight (right) over the sequential samplings.

Fig 1 shows that shell length (L) and total fresh weight (TFW) increased during the culture period, we also observe an increase in size dispersion. The one-way ANOVA by ranks and the associated Tukey HSD tests confirm a significant growth in both shell length and total fresh weight between sequential samplings, while the nonparametric dispersion tests found differences between seeding and the subsequent samplings for shell length and significant increase in dispersion for TFW between alternate samplings (see Tables A and B in S1 Appendix).

**Fig 2:**
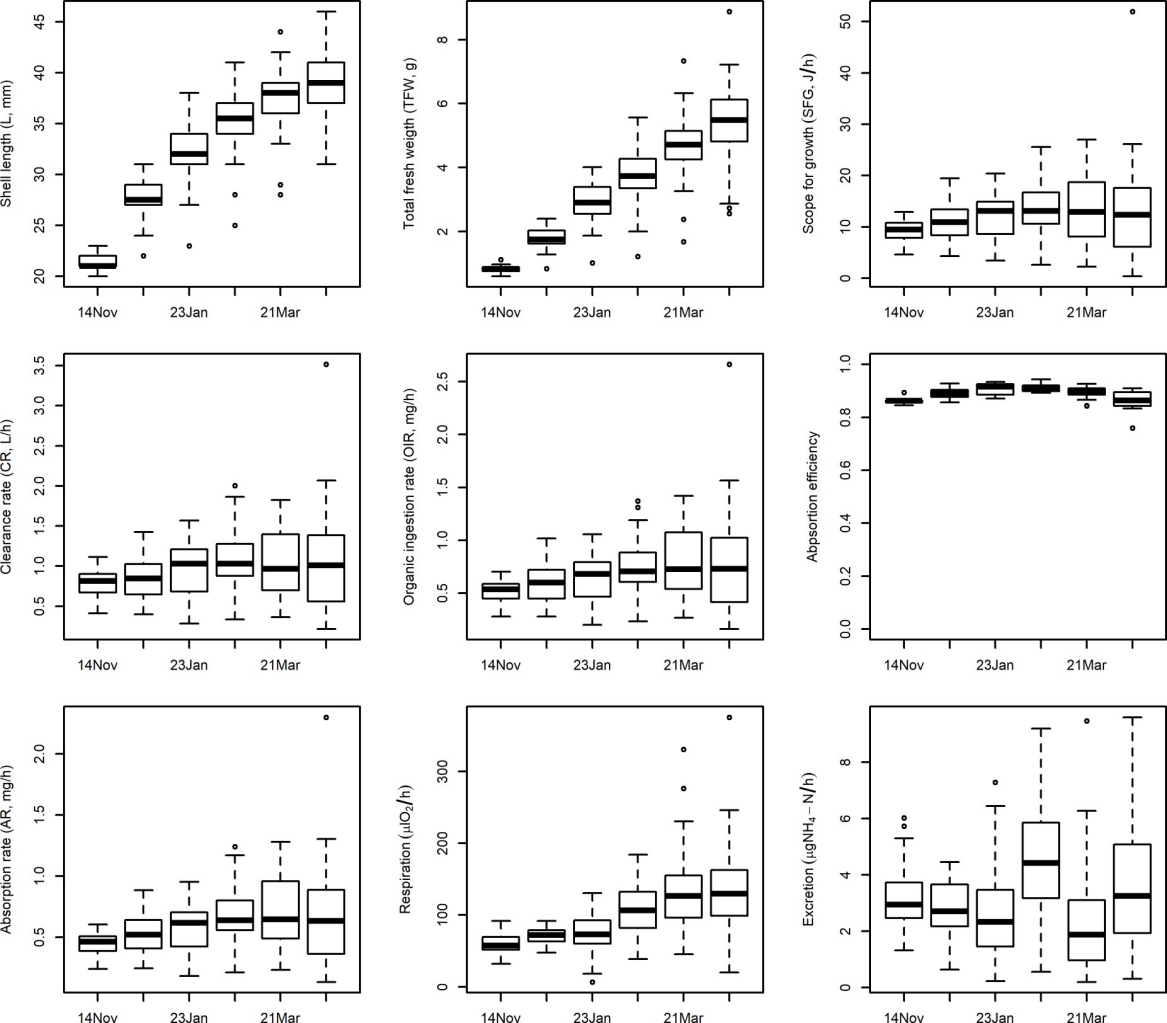
Growth and physiological performance of mussels by sampling date.

Fig 2 shows a large inter-individual variability in the physiological rates in each sampling, but we do not observe any clear trend in the phyiological performance of mussels during the experimental culture. These observations were confirmed by the homogeneity and dispersion tests. The Tukey HSD test found a significant increase in clearance rate in February, which results in significant differences in the feeding rates between the beginning and end of the experimental period, while the absorption efficiency increased up to February and decreased thereafter. The respiration rate increased significantly in February and March. The excretion rate registered a peak in February and no significant variability thereafter. Consequently, the scope for growth only reported a significant increase in February in comparison with November and remained homogeneous thereafter. On the other hand, the dispersion test detected significant increases in the variability of the physiological rates during the experiment (see Tables C and D in S1 Appendix).

**Fig 3:**
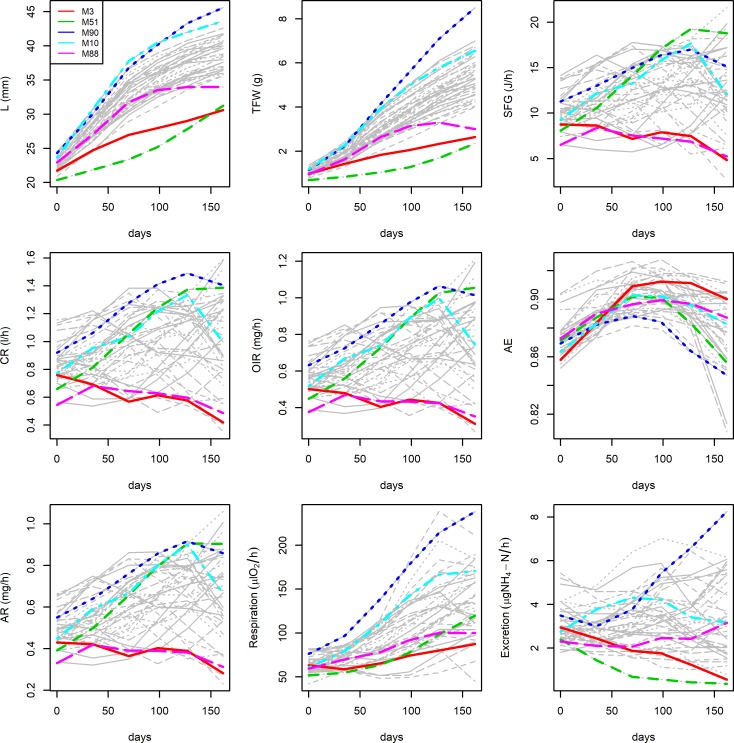
Growth curves and physiological performance for the outliers (in terms of shell length and fresh weight).

Fig 3 shows the individual growth, physiologic and metabolic curves. We have detected four outliers according with shell length (mussels 3, 51, 90, 10) and four in terms of total fresh weight (mussels 3, 51, 90, 88). If we focus on the performance of these outliers, we can see large feeding, digestion and metabolic rates, which increased over time, in the fast growing mussels (M10, M90) and low rates, which remained constant or even decreased across samplings, in mussels M3 and M88. Mussel M51 reported contradictory results, with a large SFG but slow growth in both shell and soft tissue. Outliers shall not be considered hereafter.

**Fig 4:**
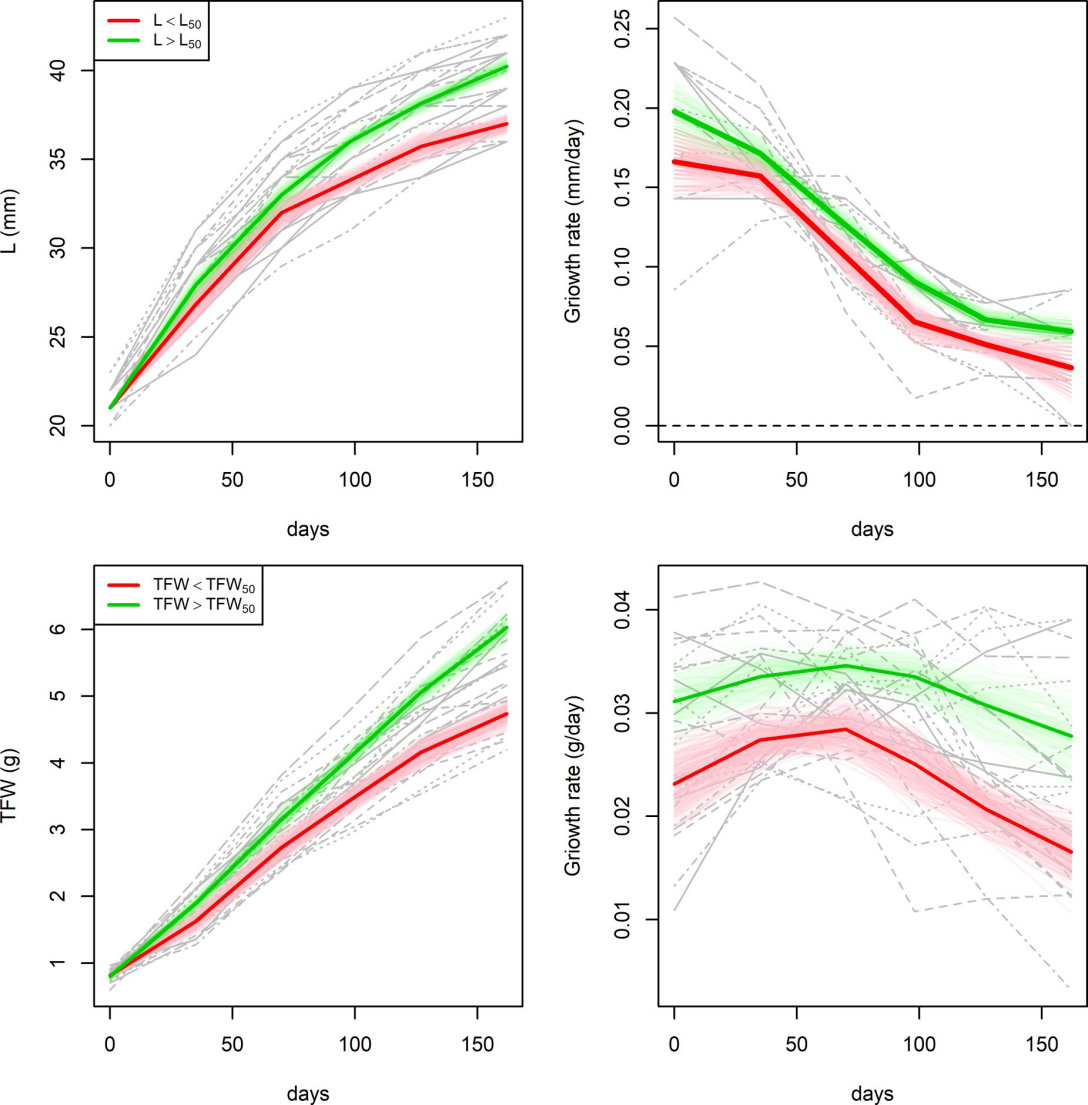
Mean (thick lines) and 95% bootstrap confidence bands of the growth curves (left) and rates (right) for mussels with L_0_ = 21mm. Mussels with final size above (fast growers, green) and below (slow growers, red) the median.

As stated above, while mussels grew significantly the physiological performance just reported a slight increase during the experimental culture. These differences between the growth and performance patterns and the low correlation found between them (functional correlation tests, p-value>0.1) advices against trying to model the growth of our mussels according with physiological rates. However, the performance of the outliers suggests that fast growing individuals may have better physiological performance than those with slow growth. This hypothesis was tested on a subsample of mussels with equal shell length (L = 21mm, n = 24) at the beginning of the physiological monitoring (14/11/2016), as differences in the initial size may introduce some bias in the interpretation of potential differences between groups in feeding, digestion and metabolic rates. Fig 4 shows growth curves and rates in terms of shell length (L) and total fresh weight (TFW) divided into two groups according with the respective median size at harvest. Mussels above and below the median reported different growth patterns for both shell length and total fresh weight (functional ANOVA, p-value < 0.005, Table 1), thus the final median provides an accurate breakpoint to classify mussels into fast and slow growers. Fast growing individuals where defined as those with final size larger than the median (L > L_50_ = , TFW > TFW_50_) and slow growing individuals as those with final size below the median (L < L_50_, TFW < TFW_50_).

**Table 1:** Functional ANOVA for comparison of scope for growth (SFG, J/h), feeding (clearance rate (CR, l/h), organic ingestion rate (OIR, mg/h)), digestion (absorption efficiency (AE), absorption rate (AR, mg/h)), metabolic (respiration (VO_2_, mlO_2_/h) and ammonia excretion rates (VNH_4_-N, μg NH_4_-N/h)) performance of mussels with final shell length (L groups) and total fresh weight (TFW groups) above and below the respective median.

	L	FW	SFG	CR	OIR	AE	AR	VO_2_	NH_4_-N
**L groups**	< .005	< .005	0.135	0.055	0.055	0.215	0.055	0.190	0.355
**TFW groups**	< .005	< .005	< .005	< .005	< .005	0.130	< .005	0.275	0.180

**Fig 5:**
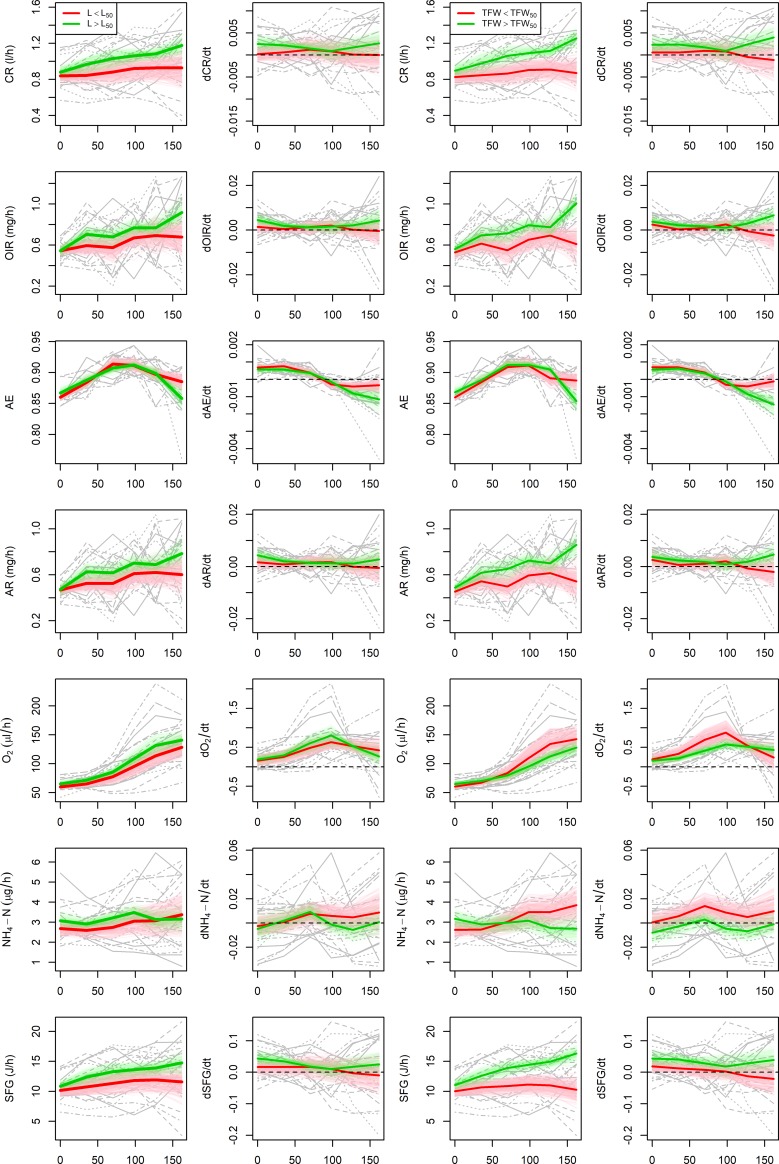
Feeding, digestion, and metabolic performance of mussel (L_0_ = 21mm) classified by final shell length (columns 1 and 2), and total fresh weight (columns 3 and 4). See acronyms in the caption of Table 1.

Fast growers reported larger feeding and digestion rates than slow growers (Fig 5, Table 1). Although significant differences between growth groups were not found (Table 1), we observe different metabolic patterns depending on the classification criteria. Mussels with fast growth in terms of shell length had larger metabolic rates during the experimental culture than slow growers, while mussels with slow growth in terms of total fresh weight had larger oxygen intake than fast growers from February onwards, which may be attributed to spawning. Consequently, we did not find any difference in SFG between groups according with shell length, but significantly larger SFG for fast growers in terms of total fresh weight

## 4 Discussion

Individual growth variability has an important role in population dynamics. Endogenous variability in bivalve growth has been mainly attributed to physiological traits [38]. Several studies have tried to identify these traits comparing the feeding, digestion and metabolic rates of fast and slow growing individuals [12–19]. All these studies are based on cross-sectional data, which measure each individual once and, consequently, may not provide enough information about the sources of growth variability [20]. As pointed out in recent studies, repeated measures of the same individuals over time, are required to distinguish individual growth variability from age or cohort effects [1,10,20].

This work introduces a new methodological framework for the analysis of bivalve growth. We have conducted sequential measurements of size and physiological performance in even-aged and even-sized mussels growing under homogeneous environmental conditions. At the end of the experiment, each mussel is characterized by a set of curves describing its growth and physiological performance. This experimental design has two important advantages over the current practice. On one hand repeated measures on the same mussel over time allow us to distinguish between changes over time within individual, i.e. growth and trends in the physiological rates, from differences between individual with respect to a baseline level [1,10,20]. On the other hand, the simultaneous monitoring of growth and physiological performance and the recently developed techniques for Functional Data Analysis allow us to identify the physiological drivers of growth variability within a homogeneous group.

Our results report an increasing dispersion in both size and physiological performance over time. Although mussels grew during the experiment, it is difficult to detect an increasing or decreasing temporal patterns in their feeding, digestion, and metabolic rates due to the large inter-individual variability. These results may seem contra-intuitive given the well stablished allometric relationships between physiological performance and bivalve size [39,40], which indicate that feeding and metabolic rates increase as individuals grow. We should point out that these relationships describe the mean performance of the population at a single time, as they are based on cross-sectional data and mean values for even-sized individuals, while this work addresses individual performance over time. Therefore, as we are dealing with different problems, we do not need to obtain similar results.

The physiological basis of inter-individual growth variability have been tested through comparison of fast and slow growing individual. The growth groups were defined according with the median size at harvest of mussels with equal shell length (L = 21mm) at the beginning of the physiological monitoring. This experimental design allows us to search for the causes of growth variability in a homogeneous group, as we measure the performance while the size differentiation is occurring, and use a single breakpoint to define the two growth groups. We have observed that mussels with fast growth were more efficient, i.e. they had higher feeding rates and lower metabolic expenditures, than those with slow growth. These results agree with the energy acquisition and the metabolic efficiency models proposed by [11], which state that fast growth may be enhanced by a faster rate of food consumption and lower metabolic costs, respectively.

Prior studies have tested the same hypothesis comparing relative physiological rates of groups with extremely fast or slow growth after size differentiation. [18] found higher size-specific clearance and absorption rates, and lower excretion rates in fast growers, but similar performance in terms of absorption efficiency and oxygen consumption. [19] found that mussels with fast growth had higher food acquisition capacity and were more energetically efficient than slow growers under both low and high food availability. Similar behaviors were reported for oysters [13,38], and clams [14–16]. Apparently, our study has found the same links between growth and physiological performance as those observed with posterior comparisons of physiological rates between groups. However, we cannot extract the same conclusions with both experimental designs. We cannot interpret differences in the physiological performance between groups that, in addition to different growth, had different sizes when feeding and metabolic rates were measured as the cause of the prior growth performance. In contrast, differences in the physiological patterns of even-sized bivalves observed during growth can be seen as drivers for growth variability.

In conclusion, the experimental design and statistical techniques proposed in this work provide valuable information for a better understanding of bivalve growth. We should highlight that Inter-individual variability in the dynamics of a homogenous population can only be detected by sequential measures of individual size and physiological performance. In addition, Functional Data Analysis provides powerful tools to summarize all the information recorded in the exhaustive sampling scheme and to test whether growth dispersion is enhanced by differences in the physiological performance.

## Supporting information

S1 Appendix Supporting information for Section 3. (DOCX)
