# Supplementary material for: Characterizing individual variability in mussel (Mytilus galloprovincialis) growth and testing its physiological drivers using Functional Data Analysis
Source: PLoS One. 2018 Oct 18;13(10):e0205981. doi: 10.1371/journal.pone.0205981 (PMC6193698; doi:10.1371/journal.pone.0205981)
Supplement: S1 Appendix — (DOCX) [file pone.0205981.s001.docx]

# **SUPPORTING INFORMATION**

**APPENDIX S1: Supporting information for Section 3**

**Table A**: Descriptive summary of Shell length (L) and total fresh weight (TFW) by sampling. Minimum (min), maximum (max), mean, standard deviation (sd) and variation coefficient (VC = sd/mean).

|  | L (mm) | | | | | TFW (g) | | | | |
| --- | --- | --- | --- | --- | --- | --- | --- | --- | --- | --- |
|  | **min** | **max** | **mean** | **sd** | **VC** | **min** | **max** | **mean** | **sd** | **VC** |
| 30/10/2016 | 20 | 20 | 20 | 0 | 0 | 0.461 | 0.911 | 0.660 | 0.089 | 0.135 |
| 14/11/2016 | 20 | 23 | 21.313 | 0.689 | 0.032 | 0.595 | 1.116 | 0.817 | 0.098 | 0.119 |
| 19/12/2016 | 22 | 31 | 27.771 | 1.871 | 0.067 | 0.837 | 2.399 | 1.777 | 0.312 | 0.176 |
| 23/01/2017 | 23 | 38 | 32.646 | 2.613 | 0.080 | 1.012 | 4.013 | 2.954 | 0.608 | 0.206 |
| 20/02/2017 | 25 | 41 | 35.188 | 2.773 | 0.079 | 1.217 | 5.560 | 3.788 | 0.804 | 0.212 |
| 21/03/2017 | 28 | 44 | 37.271 | 2.952 | 0.079 | 1.671 | 7.336 | 4.642 | 0.985 | 0.212 |
| 25/04/2017 | 31 | 46 | 38.958 | 2.910 | 0.075 | 2.558 | 8.878 | 5.407 | 1.198 | 0.222 |

**Table B:** Tukey HSD test for the ANOVA by ranks and nonparametric dispersion test for shell length (L) and total fresh weight (TFW). Significant differences (α=0.05) in bold.

|  |  | L | | TFW | |
| --- | --- | --- | --- | --- | --- |
|  | | ANOVA | Dispersion | ANOVA | Dispersion |
| DEC16-nov16 | | **1.71E-10** | **0.020** | **1.71E-10** | **0.020** |
| Jan17-nov16 | | **5.14E-13** | **2.13E-04** | **5.14E-13** | **2.13E-04** |
| feb17-nov16 | | **5.14E-13** | **7.32E-05** | **5.14E-13** | **7.32E-05** |
| mar17-nov16 | | **5.14E-13** | **1.35E-05** | **5.14E-13** | **1.35E-05** |
| Apr17-nov16 | | **5.14E-13** | **5.55E-06** | **5.14E-13** | **5.55E-06** |
| Jan17-Dec16 | | **1.02E-12** | 0.825 | **1.02E-12** | 0.825 |
| feb17-Dec16 | | **5.14E-13** | 0.684 | **5.14E-13** | 0.684 |
| mar17-Dec16 | | **5.14E-13** | 0.444 | **5.14E-13** | 0.444 |
| Apr17-Dec16 | | **5.14E-13** | 0.333 | **5.14E-13** | 0.333 |
| feb17-Jan17 | | **9.86E-07** | 1.000 | **9.86E-07** | 1.000 |
| mar17-Jan17 | | **5.68E-13** | 0.990 | **5.68E-13** | 0.990 |
| Apr17-Jan17 | | **5.14E-13** | 0.968 | **5.14E-13** | 0.968 |
| mar17-feb17 | | **2.44E-05** | 0.999 | **2.44E-05** | 0.999 |
| Apr17-feb17 | | **6.46E-13** | 0.994 | **6.46E-13** | 0.994 |
| Apr17-mar17 | | **0.010** | 1.000 | **0.010** | 1.000 |

**Table C:** Descriptive analysis (see details in the caption of Table S1) of the feeding: clearance rate (CR) and organic ingestion rate (OIR), digestion: absorption efficiency (AE), and absorption rate (AR)), metabolic rates: respiration (VO_2_) and ammonia excretion (VNH_4_-N), and scope for growth (SFG) of mussels across samplings.

|  |  | min | max | mean | sd | VC |
| --- | --- | --- | --- | --- | --- | --- |
| CR (l/h) | 14/11/2016 | 0.411 | 1.114 | 0.802 | 0.157 | 0.196 |
|  | 19/12/2016 | 0.400 | 1.425 | 0.853 | 0.252 | 0.295 |
|  | 23/01/2017 | 0.284 | 1.569 | 0.943 | 0.332 | 0.352 |
|  | 20/02/2017 | 0.334 | 2.004 | 1.076 | 0.350 | 0.325 |
|  | 21/03/2017 | 0.364 | 1.825 | 1.045 | 0.426 | 0.408 |
|  | 25/04/2017 | 0.214 | 3.516 | 1.039 | 0.592 | 0.569 |
| OIR (mg/h) | 14/11/2016 | 0.281 | 0.703 | 0.528 | 0.100 | 0.189 |
|  | 19/12/2016 | 0.280 | 1.019 | 0.602 | 0.179 | 0.298 |
|  | 23/01/2017 | 0.201 | 1.056 | 0.635 | 0.218 | 0.343 |
|  | 20/02/2017 | 0.235 | 1.373 | 0.741 | 0.241 | 0.325 |
|  | 21/03/2017 | 0.268 | 1.421 | 0.794 | 0.324 | 0.408 |
|  | 25/04/2017 | 0.162 | 2.661 | 0.764 | 0.445 | 0.583 |
| AE | 14/11/2016 | 0.846 | 0.894 | 0.863 | 0.012 | 0.014 |
|  | 19/12/2016 | 0.857 | 0.927 | 0.890 | 0.020 | 0.022 |
|  | 23/01/2017 | 0.871 | 0.934 | 0.907 | 0.020 | 0.022 |
|  | 20/02/2017 | 0.893 | 0.944 | 0.911 | 0.014 | 0.015 |
|  | 21/03/2017 | 0.844 | 0.926 | 0.895 | 0.022 | 0.024 |
|  | 25/04/2017 | 0.760 | 0.910 | 0.866 | 0.034 | 0.039 |
| AR (mg/h) | 14/11/2016 | 0.241 | 0.607 | 0.456 | 0.089 | 0.194 |
|  | 19/12/2016 | 0.247 | 0.885 | 0.535 | 0.159 | 0.297 |
|  | 23/01/2017 | 0.185 | 0.954 | 0.575 | 0.196 | 0.341 |
|  | 20/02/2017 | 0.214 | 1.242 | 0.674 | 0.217 | 0.322 |
|  | 21/03/2017 | 0.233 | 1.280 | 0.710 | 0.286 | 0.403 |
|  | 25/04/2017 | 0.135 | 2.300 | 0.661 | 0.382 | 0.579 |
| VO_2_ (mlO_2_/h) | 14/11/2016 | 32.04 | 91.84 | 60.31 | 13.112 | 0.217 |
|  | 19/12/2016 | 47.63 | 91.88 | 71.44 | 10.968 | 0.154 |
|  | 23/01/2017 | 6.72 | 130.37 | 73.87 | 25.736 | 0.348 |
|  | 20/02/2017 | 38.47 | 184.13 | 107.83 | 34.867 | 0.323 |
|  | 21/03/2017 | 45.19 | 330.53 | 134.48 | 55.958 | 0.416 |
|  | 25/04/2017 | 19.79 | 375.35 | 137.44 | 55.511 | 0.404 |
| NH_4_-N (µg NH_4_-N/h) | 14/11/2016 | 1.310 | 6.025 | 3.174 | 1.017 | 0.321 |
|  | 19/12/2016 | 0.630 | 4.455 | 2.846 | 0.949 | 0.334 |
|  | 23/01/2017 | 0.220 | 7.285 | 2.576 | 1.471 | 0.571 |
|  | 20/02/2017 | 0.550 | 9.200 | 4.693 | 2.017 | 0.430 |
|  | 21/03/2017 | 0.193 | 9.468 | 2.297 | 1.834 | 0.798 |
|  | 25/04/2017 | 0.300 | 9.600 | 3.573 | 2.240 | 0.627 |
| sfg (J/h) | 14/11/2016 | 4.584 | 12.905 | 9.406 | 2.023 | 0.215 |
|  | 19/12/2016 | 4.274 | 19.427 | 11.048 | 3.674 | 0.333 |
|  | 23/01/2017 | 3.440 | 20.394 | 11.943 | 4.354 | 0.365 |
|  | 20/02/2017 | 2.633 | 25.575 | 13.537 | 4.956 | 0.366 |
|  | 21/03/2017 | 2.247 | 27.007 | 13.899 | 6.340 | 0.456 |
|  | 25/04/2017 | 0.375 | 51.956 | 12.822 | 8.950 | 0.698 |

**Table D:** Tukey HSD test for the ANOVA by ranks and nonparametric dispersion test for the feeding, digestion and metabolic performance of mussels (see details in the caption of Table S3).

|  |  | CR | | OIR | | AE | | AR | | VO_2_ | | NH_4_-N | | SFG | |
| --- | --- | --- | --- | --- | --- | --- | --- | --- | --- | --- | --- | --- | --- | --- | --- |
|  |  | ANOVA | Dispersion | ANOVA | Dispersion | ANOVA | Dispersion | ANOVA | Dispersion | ANOVA | Dispersion | ANOVA | Dispersion | ANOVA | Dispersion |
| DEC16-NOV16 | | 0.985 | 0.518 | 0.771 | 0.399 | 0.977 | **0.029** | 0.583 | 0.400 | 0.690 | 1.000 | 0.962 | 1.000 | 0.673 | 0.399 |
| JAN17-NOV16 | | 0.439 | **0.044** | 0.388 | 0.073 | **3.0E-08** | **0.004** | 0.145 | 0.054 | 0.481 | 0.460 | 0.580 | 0.617 | 0.199 | 0.067 |
| FEB17-NOV16 | | **0.028** | **0.038** | **0.002** | 0.044 | **1.8E-11** | 0.760 | **6.0E-04** | **0.031** | **2.6E-08** | **0.010** | 0.100 | **0.001** | 0.034 | **0.027** |
| MAR17-NOV16 | | **0.022** | **1.9E-04** | **0.000** | **1.6E-05** | **5.7E-13** | **0.017** | **1.5E-04** | **1.9E-05** | **5.7E-13** | **1.7E-07** | **0.003** | 0.185 | 0.003 | **4.6E-05** |
| APR17-NOV16 | | **0.005** | **1.5E-09** | **4.9E-05** | **1.0E-10** | **5.5E-13** | **3.0E-11** | **7.0E-06** | **6.1E-10** | **5.7E-13** | **1.1E-06** | **1.5E-10** | **2.0E-04** | **0.001** | **1.3E-09** |
| JAN17-DEC16 | | 0.848 | 0.839 | 0.991 | 0.964 | **1.5E-06** | 0.991 | 0.964 | 0.937 | 1.000 | 0.316 | 0.967 | 0.681 | 0.965 | 0.957 |
| FEB17-DEC16 | | 0.157 | 0.814 | 0.126 | 0.917 | **1.6E-09** | 0.521 | 0.113 | 0.868 | **4.4E-05** | **4.1E-03** | 0.484 | **0.001** | 0.624 | 0.849 |
| MAR17-DEC16 | | 0.132 | 0.072 | 0.046 | 0.026 | **5.7E-13** | 1.000 | 0.051 | 0.029 | **6.6E-13** | **4.4E-08** | **0.047** | 0.227 | 0.216 | 0.050 |
| APR17-DEC16 | | **0.043** | **1.4E-05** | 0.009 | **3.8E-06** | **5.7E-13** | **3.3E-04** | **0.006** | **1.6E-05** | **5.8E-13** | **3.2E-07** | **1.9E-08** | **3.0E-04** | 0.112 | **2.5E-05** |
| FEB17-JAN17 | | 0.814 | 1.000 | 0.401 | 1.000 | 0.812 | 0.190 | 0.505 | 1.000 | **0.000** | 0.598 | 0.926 | 0.095 | 0.972 | 1.000 |
| MAR17-JAN17 | | 0.771 | 0.633 | 0.196 | 0.206 | **0.002** | 0.998 | 0.321 | 0.273 | **1.3E-12** | **8.4E-04** | 0.286 | 0.974 | 0.700 | 0.328 |
| APR17-JAN17 | | 0.502 | **0.002** | 0.055 | **1.6E-04** | **4.3E-05** | **0.003** | 0.071 | **8.8E-04** | **6.3E-13** | **0.003** | **1.4E-06** | **0.048** | 0.499 | **8.7E-04** |
| MAR17-FEB17 | | 1.000 | 0.664 | 0.998 | 0.291 | 0.083 | 0.414 | 1.000 | 0.379 | **0.007** | 0.135 | 0.857 | 0.425 | 0.989 | 0.526 |
| APR17-FEB17 | | 0.997 | **0.003** | 0.932 | **3.5E-04** | **0.007** | **8.0E-08** | 0.921 | 0.002 | **0.002** | 0.285 | **1.4E-04** | 1.000 | 0.936 | **0.003** |
| APR17-MAR17 | | 0.999 | 0.212 | 0.995 | 0.256 | 0.958 | **6.6E-04** | 0.979 | 0.398 | 0.999 | 0.999 | 0.016 | 0.268 | 1.000 | 0.315 |
